# Comorbidity characteristics of multiple myeloma patients diagnosed in Finland 2005–2016

**DOI:** 10.1007/s00277-022-04959-9

**Published:** 2022-09-13

**Authors:** Iiro Toppila, Kai Kysenius, Tatu Miettinen, Mariann Ida Lassenius, Juha Lievonen, Pekka Anttila

**Affiliations:** 1 Medaffcon Oy, Espoo, Finland; 2 Takeda Oy, Helsinki, Finland; 3 Department of Hematology, University of Helsinki and Helsinki University Hospital, Helsinki, Finland

**Keywords:** Multiple myeloma, Comorbidities, Real-world data, Hematology, Oncology, Cardiovascular disease, Secondary malignancy, Finland

## Abstract

Multiple myeloma (MM) patients are predominantly elderly with comorbidities that have an impact on patient mortality and treatment decisions. We previously reported the patient characteristics and overall survival outcomes of the Finnish MM cohort diagnosed between 2005 and 2016 in a nationwide retrospective registry study comprising 3,851 adults. Here, we report detailed comorbidity characteristics for this real-world Finnish MM population at cohort entry and during follow-up. Data on diagnoses and causes of death were obtained from Finnish healthcare data registries and interrogated using various multistate time-to-event models. In the year preceding MM diagnosis, comorbidities (as per Charlson Comorbidity Index definition) were recorded in 38.0% of the cohort, of which 27.9% presented with pre-existing cardiovascular disease (CVD) and 4.8% had suffered a major adverse cardiac event (MACE). At 2 years post-MM diagnosis, cumulative incidence for CVD and MACE more than doubled to 57.1% and 11.4%, respectively, and only 31.9% of the cohort remained CVD-free. Prevalent secondary malignancies were recorded in 16.8% of the patient population at MM diagnosis, with cumulative incidence increasing steadily to 27.5% at 2 years and 33% at 5 years post-diagnosis. The main cause of mortality attributed to MM, CVD, secondary malignancy, or other causes remained stable throughout the follow-up, at an average of 74.2%, 9.4%, 9.8%, and 6.5%, respectively. Prevalence of CVDs and secondary malignancies is high in Finnish patients at MM diagnosis, with older male patients suffering from higher MACE and mortality risk. Proper recording and management of comorbidities alongside novel treatments remain crucial for optimal MM management.

**Supplementary Information:**

The online version contains supplementary material available at 10.1007/s00277-022-04959-9.

## Introduction

Multiple myeloma (MM) primarily affects the elderly with most cases detected between 65 and 75 years of age. Global age-standardized incidence rate is reported as 2.1 per 100,000 people, varying between 0.54 and 5.3, with the highest rates in Oceania and North America [1, 2]. In Finland, age-standardized incidence rate is 3 per 100,000 people with more than 300 people diagnosed annually with MM, approximately 15% of all yearly malignant haematological cancer diagnoses [3]. Overall survival (OS) estimates are highly variable ranging from 2 to 3 years to over a decade depending on patient age, availability of novel therapeutics, whether autologous stem cell transplant (ASCT) is possible and overall health concerns such as comorbidities [4, 5].

Most patients with MM are elderly, and many are affected by significant heterogenous age-related comorbidities that can affect treatment decisions. Common comorbidities include cardiovascular diseases (CVDs), secondary malignancies, and infections [6]. These common comorbidities present a challenge for patients with MM when assessing optimal treatment course and patient outcomes, as most MM drugs can have adverse effects, especially on the cardiovascular system [7, 8]. Whilst ASCT remains the single most effective treatment for fit patients under 65–75 years of age (depending on country and treatment practices), the treatment options have fortunately increased [9]. Within the past two decades, MM treatments have transformed with the development of immunomodulatory drugs (IMIDs), proteosome inhibitors (PIs), and targeted monoclonal antibody therapies, administered either as mono- or combination therapies [10, 11].

Well-executed clinical trials can provide robust data and statistical power to evaluate drug efficacy in a well-curated study population, but often fail to represent the full spectrum of patients affected by a given disease or condition [12]. Especially for indications primarily affecting the elderly, such as MM, real-world evidence (RWE) epidemiological studies can provide an improved understanding of comorbidities in the true MM population outside of clinical trials run under strict criteria [12]. Several clinical trials have raised concerns for modestly increased risk of secondary malignancies related to several IMIDs, such as lenalidomide, and antibody-based therapies; however, the overall benefits likely outweigh the risks, and based on International Myeloma Working Group (IMWG) recommendations, should not impact the current therapeutic decision-making process [13–16].

To date, only few recent epidemiological studies have explored the comorbidity characteristics of patients with MM at either clinic or national level with attempts to elucidate prognostic predictors of survival within these cohorts. A recent Danish nationwide MM study reported an increased comorbidity burden in patients with MM relative to controls, and increased mortality in patients with MM with one or more recorded comorbidities [17]. Other common findings include increased CVD comorbidity load in older patients, especially coronary disease and heart failure [18]. However, most MM-centric RWE studies have largely focused on particular treatment options [19], individual comorbidities or disease classes (mainly on CVDs) [13, 14], or survival measures [20], thus not providing a comprehensive understanding of prevalent comorbidities as well as incident comorbidity load during follow-up. In this study, we aim to provide a comprehensive understanding of comorbidity burden and prognostic factors within the Finnish MM population. Our recent study [5] describes the clinical characteristics and survival of 3,851 Finnish patients with MM diagnosed between 2005 and 2016, highlighting continuous improvements in overall survival (OS) throughout the observation period. This RWE study provides an in-depth look into longitudinal comorbidity characteristics within the Finnish real-world MM population and the occurrence of severe comorbidities accounting for competing risk of mortality. Additionally, causes of death are assessed in more detail.

## Methods

### Ethics clearance and data sources

The MM cohort and associated data for this retrospective real-world evidence (RWE) study were based on the information requested from the national Finnish Care Register for Health Care (HILMO), Statistics Finland, and Finnish Social Insurance Institution (SII). The study was approved by each registered holder.

### Cohort formation

Cohort formation is described in detail in Toppila et al., AOHE 2021 [5]. Briefly, patients diagnosed with C90.0 (multiple myeloma; ICD-10 diagnosis classification) from HILMO data between 1.1.2005 and 31.12.2016 in Finland were included in the cohort. Patients were excluded if they were as follows: MM diagnosed prior to 1.1.2005; not a citizen of Finland; three or fewer C90.0 diagnosis codes (potential misdiagnosis); no treatment within the first year; recipient of ASCT before 1.1.2005 or diagnosis of MM; or age at diagnosis below 18 years. Treatment initiation time was set as the date of specialty reimbursement, first-ever purchase for MM-specific drug, or date for ASCT.

### Data analysis

All diagnosis codes recorded in the HILMO system as ICD-10 International Classification of Diseases, Tenth Revision (ICD-10) codes were gathered for each MM patient. Statistics Finland records were accessed to obtain data on the main and immediate causes of death. In this study, diagnoses and comorbidities are reported in three forms: Firstly, we report comorbidities grouped into CVDs, major adverse cardiac events (MACE), and malignancies (Table 1). We acknowledge that MACE is subject to heterogenous use, as it lacks a standard definition [21]. Our MACE composite is defined based on the clinical endpoints as listed in Table 1, inclusive of cardiovascular death, myocardial infarction, cardiac operations, ischemic stroke, and hospitalization due to heart failure. Secondly, we report all recorded ICD-10 diagnosis codes (Supplementary Table 1). Lastly, we report comorbidities based on diseases included in the Charlson comorbidity index (CCI) [22, 23], with a minor modification: metastatic cancer diagnoses (C77–C80) were excluded if no primary cancer diagnosis was not recorded at any point in the data (Supplementary Table 2).

**Table 1:** Grouping of comorbidity diagnosis codes for CVDs and malignancies

Diagnosis group	ICD-10 diagnoses and procedure codes	Notes
CVD	I10-I79	Any heart or vasculature-associated comorbidity
Mace (major adverse cardiac event)	CVD death (Any death, with the main cause of death or immediate cause of death marked as ICD-10: I00-I99) Heart failure hospitalization (I50* – **only** as the main diagnosis code for hospitalization) Myocardial infarction (I21; I22; I23.1; I23.2; I23.3; I23.4; I23.5) Ischemic stroke (I63; I64; I65.1; I65.2; I65.8; I65.9; I66) **Procedures**: Coronary artery bypass surgery, stents: FNA*, FNC*, FNE*, FN2	MACE-like composite endpoint [21]
Malignancy	C00-97 (excluding C90.0), D09.0–1, D32-33, D41-43, D45-46, D76 Note – D47.2 excluded; Metastatic cancer diagnoses (C77-C80) were excluded if no primary cancer diagnosis was not recorded at any point in the data (as in Finnish clinical practice especially C79.5 among other metastatic cancer diagnoses are routinely used with bone manifestations and/or extramedullary plasmacytomas, whereas it is uncommon in Finland to record only metastatic cancer without further records of the primary tumor)	ICD-10 data collected from HILMO data, based on list from Finnish Cancer Registry [3]

### Statistical analysis

The cohort was described at MM diagnosis by the proportion of each reported ICD-10 diagnosis code (prevalence ≥ 5% in the cohort) up to a year before the MM index date (date of study entry).

**Fig. 1:**
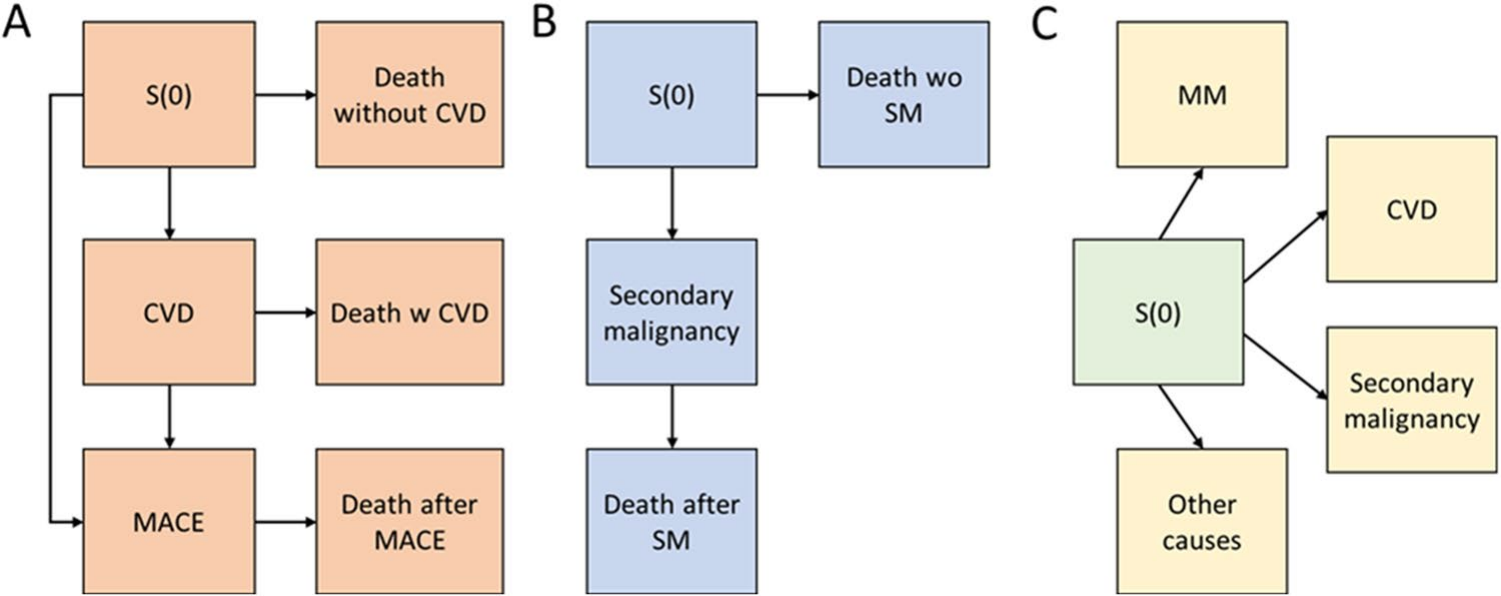
Multistate model diagram for CVD (A), secondary malignancy (B) and the main cause of mortality (C). Patients move from initial state S(0) to either disease states following diagnosis (CVD or MACE for A; secondary malignancy for B), or death with or without additional comorbidities, which are based on recorded diagnosis (A and B) or recorded primary cause of death (C). Treated or resolved CVD, MACE, or secondary malignancy does not return the patient to the initial state S(0). A patient can enter the study (MM diagnosis) at S(0) or any disease state. CVD = cardiovascular disease; MM = multiple myeloma; SM = secondary malignancy; S(0) = Neutral initial state

The CVD comorbidity, secondary malignancies, and cause-specific mortality were analyzed in multistate time-to-event models (see Fig. 1 for multistate specifications) estimating Aalen-Johansen state probabilities, i.e., the proportion of patients in different groups in any given time, and corresponding cumulative incidences as a function of time accounting for patient censoring (at end of follow-up 31.12.2016). The following predictors of the event (CVD, secondary malignancy, or death) were included as covariates in corresponding multistate Cox models: age at MM diagnosis, sex, ASCT (time-varying covariate), and diagnosis year, and the models were stratified by CCI category corresponding to Toppila et al. [5]. Models were set to have shared coefficients for transitions to any of the death states, and for MACE state. I.e., covariates, were set to have the same effect irrespective of the previous state, for example in transition from CVD-free state (S0) to MACE and from CVD to MACE (Fig. 1).

## Results

### Study population

Comorbidity data were included from 3851 patients with MM diagnosed between 1.1.2005 and 31.12.2016 in Finland. For a detailed description of the study cohort, please refer to the “Methods” section and Toppila et al., 2021 AOHE [5].

### Healthcare visit-related diagnoses at study entry

Comorbidity analyses were based on ICD-10 codes recorded in HILMO (specialty care) up to one year before the initial MM diagnosis (index date). The full list of diagnoses is presented in Supplementary Table 1. In short, several common diagnoses, such as other anaemias (D64), other special examinations (Z01), and an elevated erythrocyte sedimentation rate and abnormality of plasma viscosity (R70) fit conditions and symptoms related to MM and may be connected to investigations prior to MM diagnosis confirmation [24]. Similarly, pain states such as dorsalgia (M54) and infections such as pneumonia (J18) are commonly reported in MM [25]. Cardiovascular disease–related comorbidities (I10, I48, I25, and I50) that may affect treatment choices at diagnosis are relatively common in patients with MM and are discussed in more detail in later chapters.

### CCI-index comorbidity characteristics at study entry

Comorbidity characteristics are often grouped according to the Charlson Comorbidity Index (CCI), which comprises 19 diseases used to produce a composite score for clinical prognosis and comorbidity adjustments in health services research [22, 23, 26]. To provide a better comparison to past and future studies, we reclassified comorbidities according to the CCI and compared our CCI-based comorbidity listing to a similar MM cohort collated by Gregersen and colleagues in Denmark, which included 2190 Danish patients with MM diagnosed between 2005 and 2012 (Supplementary Table 2)[17].

CCI-listed comorbidities were recorded in 1457 (38.0%) Finnish newly diagnosed patients with MM, which closely corresponds to the Danish MM cohort (40.9%). The most prominent disease categories in the Finnish MM cohort were secondary malignancies (12.5%), renal disease (7.9%; includes all severities), diabetes without chronic complications (6.2%), congestive heart failure (5.7%), and chronic pulmonary disease (4.8%). When comparing the two cohorts, statistically significant differences (*P* < 0.05) were seen in 10 CCI classes: in the Finnish cohort, less patients had a myocardial infarction, peripheral vascular disease, cerebrovascular disease, peptic ulcer disease and diabetes with chronic complications, and more patients had, diabetes without chronic complications, hemiplegia or paraplegia, and renal disease (Supplementary Table 2). However, absolute differences were in most cases small, and the clinical significance of these findings remains unclear, although some country-specific data suggest that country-specific differences do exist, especially in the context of CVD, diabetes, and malignancies [27, 28]. However, these differences may also be contributable to comorbidity recording practices.

### Incident cardiovascular diseases during follow-up

As stated previously, CVDs are common in patients with MM and have a direct impact on treatment decisions, as commonly prescribed MM drugs have differing profiles of cardiotoxic effects [8]. Hence, understanding the characteristics of the real-world MM population is helpful in determining optimal patient treatment and can facilitate improved communication between treating oncologists and cardiologists [7].

**Fig. 2:**
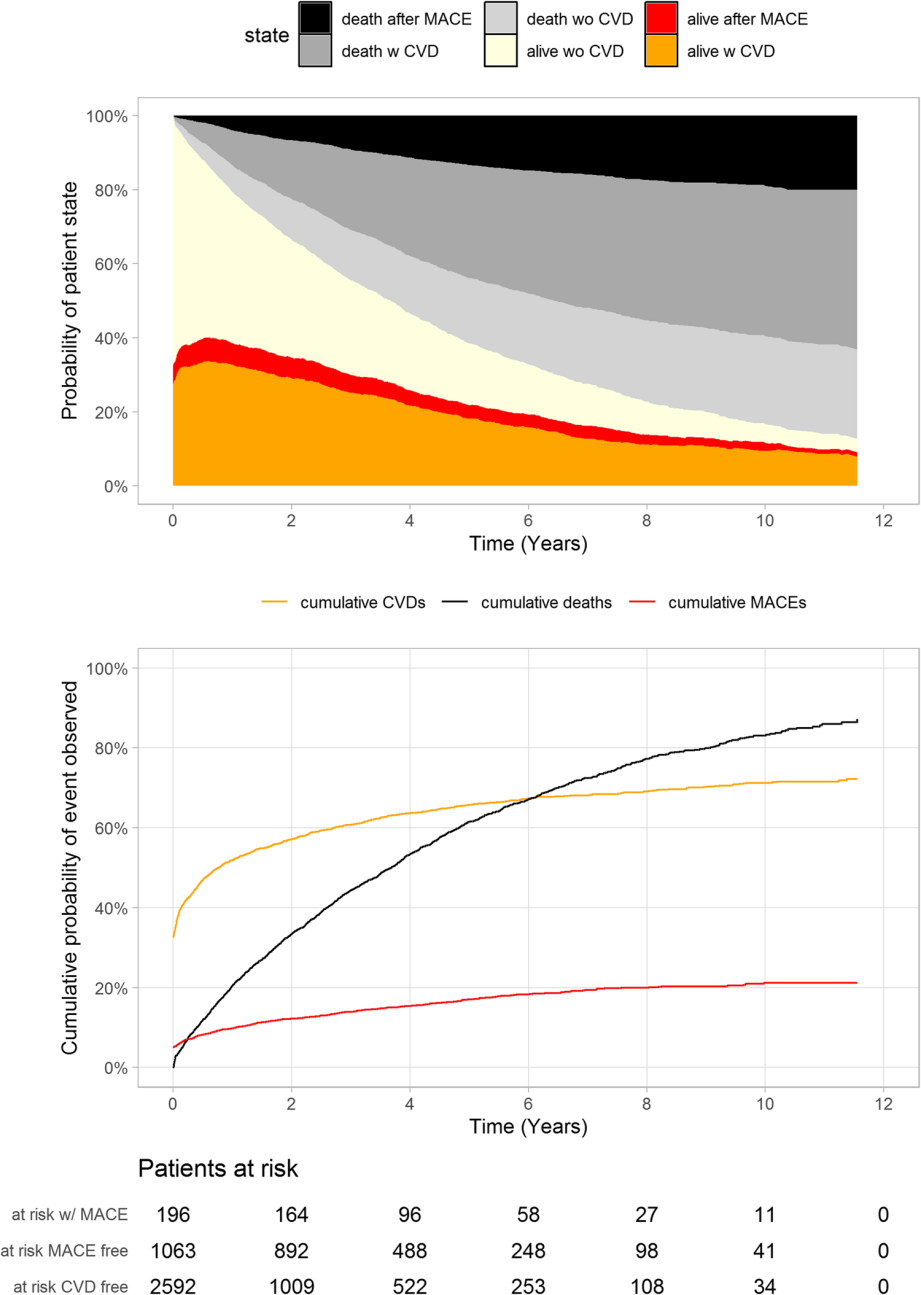
Timing of incident CVD and MACE events after MM diagnosis. Data is presented both as a probability of patient state (above) and cumulative probability of an observed event (below). CVD = cardiovascular disease (as defined in Methods); MACE = major adverse cardiac event

Based on ICD-10 codes for all diseases involving the heart and vasculature I10-I79 (as per Methods), 27.9% of patients with MM had a prevalent CVD diagnosis at study entry, with cumulative incidence almost doubling to 55% at 2 years post-diagnosis (Fig. 2). Additionally, 5.1% of MM population had suffered a MACE before MM diagnosis, with the cumulative incidence of new MACE doubling in 2 years and reaching 20% at 8 years (Fig. 2**).** The proportion of patients alive and CVD-free diminished to 40.8%, 31.9%, and 16.7% at 1, 2, and 5 years post-MM diagnosis, respectively, highlighting the substantial and increasing burden of CVD on patients with MM during follow-up and successive lines of treatment (Fig. 2). Majority of patients with MM also die with CVD or after MACE, cementing the importance of CVD considerations for patient outcomes.

**Fig. 3:**
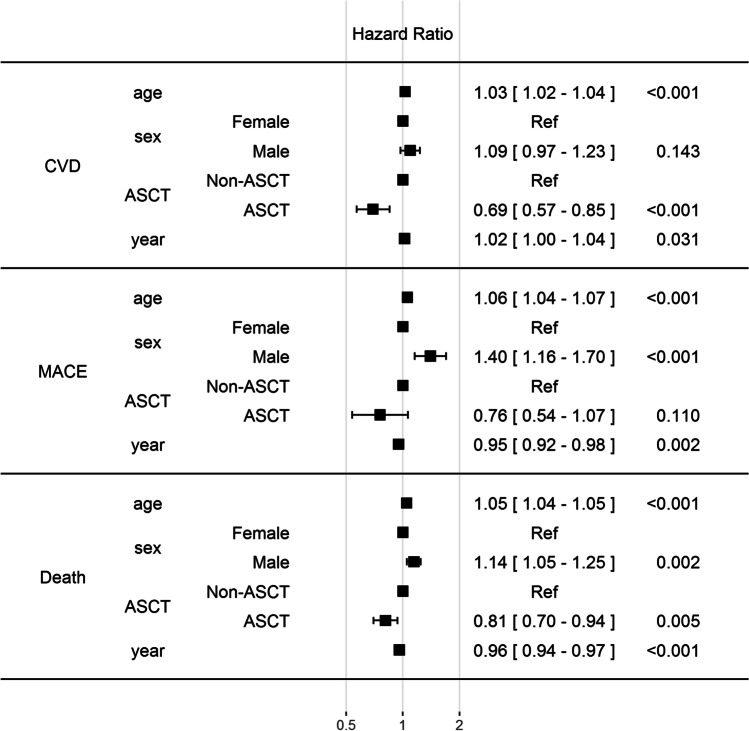
Forest plot of multistate cox proportional hazards risk estimates for CVD, MACE, and death**.** CVD = cardiovascular disease; MACE = major adverse cardiac event; ASCT = autologous stem cell transplant. See methods for further details

We assessed the significance of covariates (age at diagnosis, sex, ASCT, and year of diagnosis) on the risk of CVD, MACE, and subsequent death using multistate Cox hazard analyses (Fig. 3). Our data show that CVD, MACE, and risk of death is increased in older patients but, interestingly, more CVD diagnoses but fewer MACE are recorded during later years of diagnosis. Patients that have received ASCT are at decreased risk of CVD or death, without an effect on MACE occurrence. Male patients have a significantly higher risk for MACE and death. Decreased risk of death (0.96) at the later year of diagnosis is in line with our previous study, indicating improved MM patient survival [5]. Overall, these data confirm that CVDs and mortality are high in patients with MM, but follow the positive trend of CVD-related management and mortality improvements seen in the general Finnish population during the past decades [29].

### Incidence and types of secondary malignancies during follow-up

Novel therapeutics have improved the OS of patients with MM, but the concomitant risk of secondary malignancy development has also increased [30]. Immunological responses are severely impacted in MM throughout the disease process and medications, especially IMIDs, thus malignancies remain an important concern for patients with MM and can affect treatment decisions [16].

**Fig. 4:**
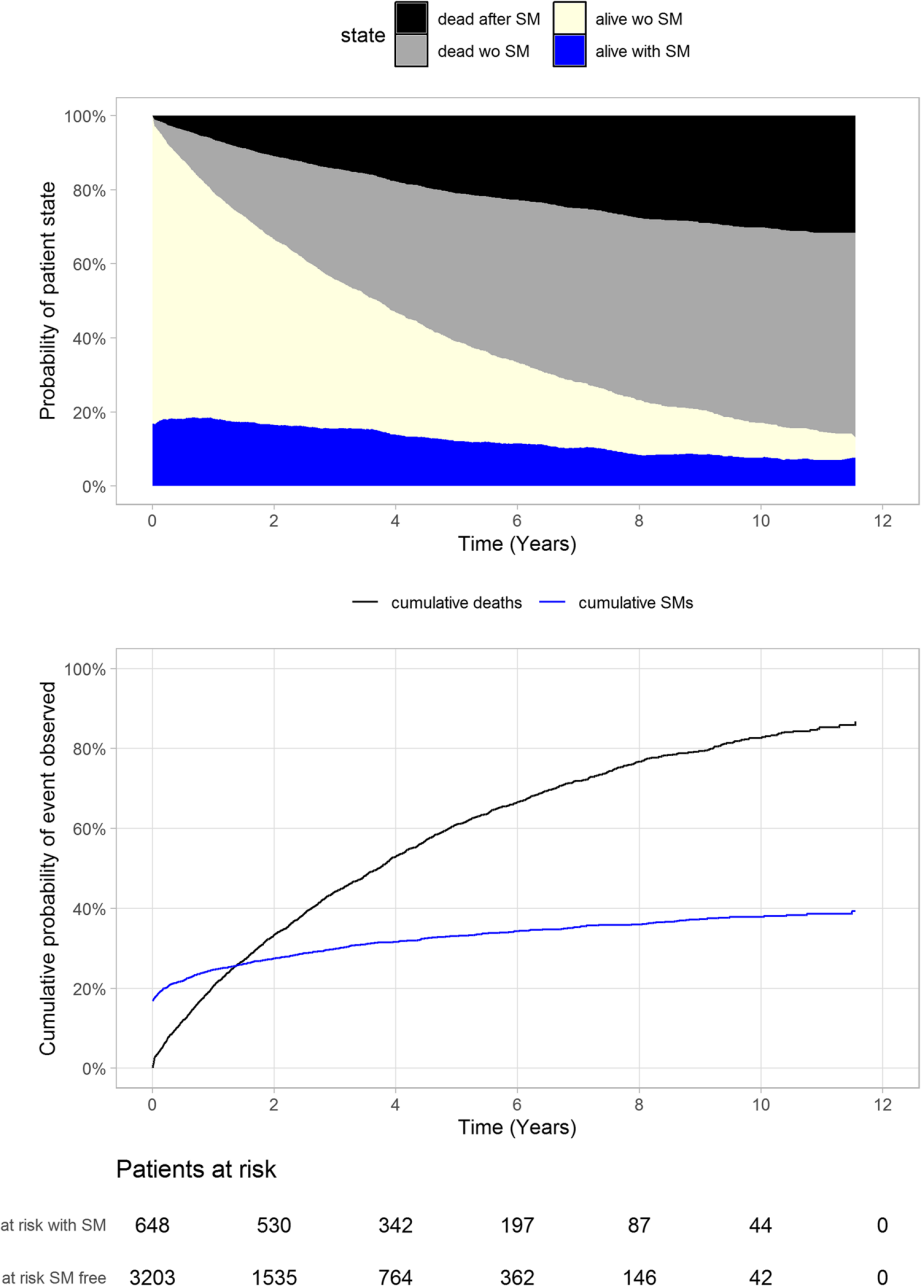
Proportion of patients with MM dying with or without secondary malignancies during follow-up. Data is presented both as a probability of patient state (above) and cumulative probability of an observed event (below). SM = secondary malignancies; wo = without

At study entry, 16.8% of the Finnish MM cohort had a prevalent malignancy other than MM or monoclonal gammopathy of undetermined significance (MGUS). The cumulative incidence of secondary malignancies increased to 24.6%, 27.5.5%, and 33.0% at 1, 2, and 5 years after MM diagnosis, respectively (Fig. 4). Similar to CVDs, the proportion of surviving patients with MM that remained free of secondary malignancies dropped sharply within the first years after MM diagnosis, falling to 50.1% and 26.9% 2 and 5 years after diagnosis, respectively (Fig. 4). The percentage of patients with MM dying with a secondary malignancy (regardless of the primary cause of death) remained stable throughout the follow-up (31–37%).

The full list of cancer diagnoses at study entry are listed in Supplementary Table 3 and the most common incident secondary malignancies in Supplementary Table 4. The cancer types in the prevalent and incident setting remain largely the same with a few notable exceptions. Prostate and breast cancer are the two most common malignancies preceding the diagnosis of MM. Bone and cartilage tumors (C41) are less frequent post-MM, whereas lymphoid (C91) and myeloid leukemias (C92), and skin cancers (C44) become more common post-MM (Supplementary Table 4).

**Fig. 5:**
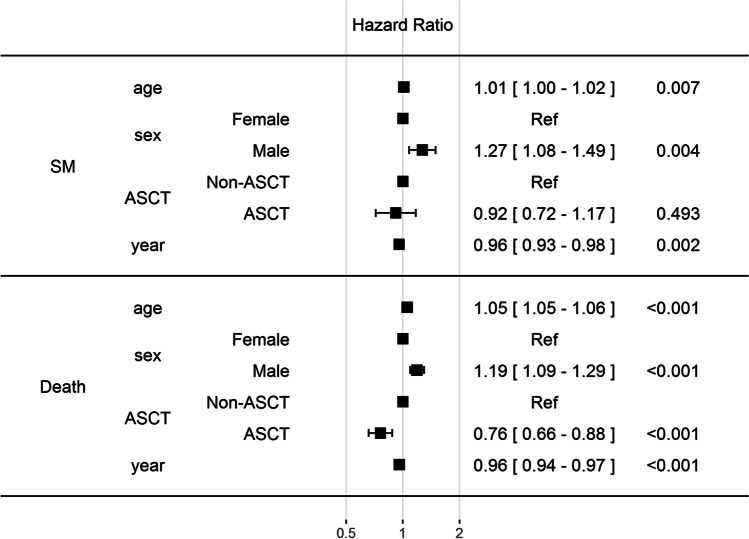
Forest plot of multistate cox proportional hazards risk estimates for secondary malignancies (SM) and SM-associated death**.** SM = secondary malignancy; ASCT = autologous stem cell transplant. See methods for further details.

We assessed the significance of covariates (age at diagnosis, sex, ASCT, and year of diagnosis) on the risk of secondary malignancies and subsequent death using multistate Cox hazard analyses (Fig. 5). Our data show that older males are at increased risk of secondary malignancies (*p* = 0.004) and death (*p* < 0.001), whereas later year of diagnosis decreases secondary malignancy risk (*p* = 0.002) and death (*p* < 0.001). As expected, ASCT decreases the risk of death (*p* =  < 0.001) without an effect on secondary malignancies (*p* = 0.493).

### Effects of comorbidities on MM patient mortality

**Fig. 6:**
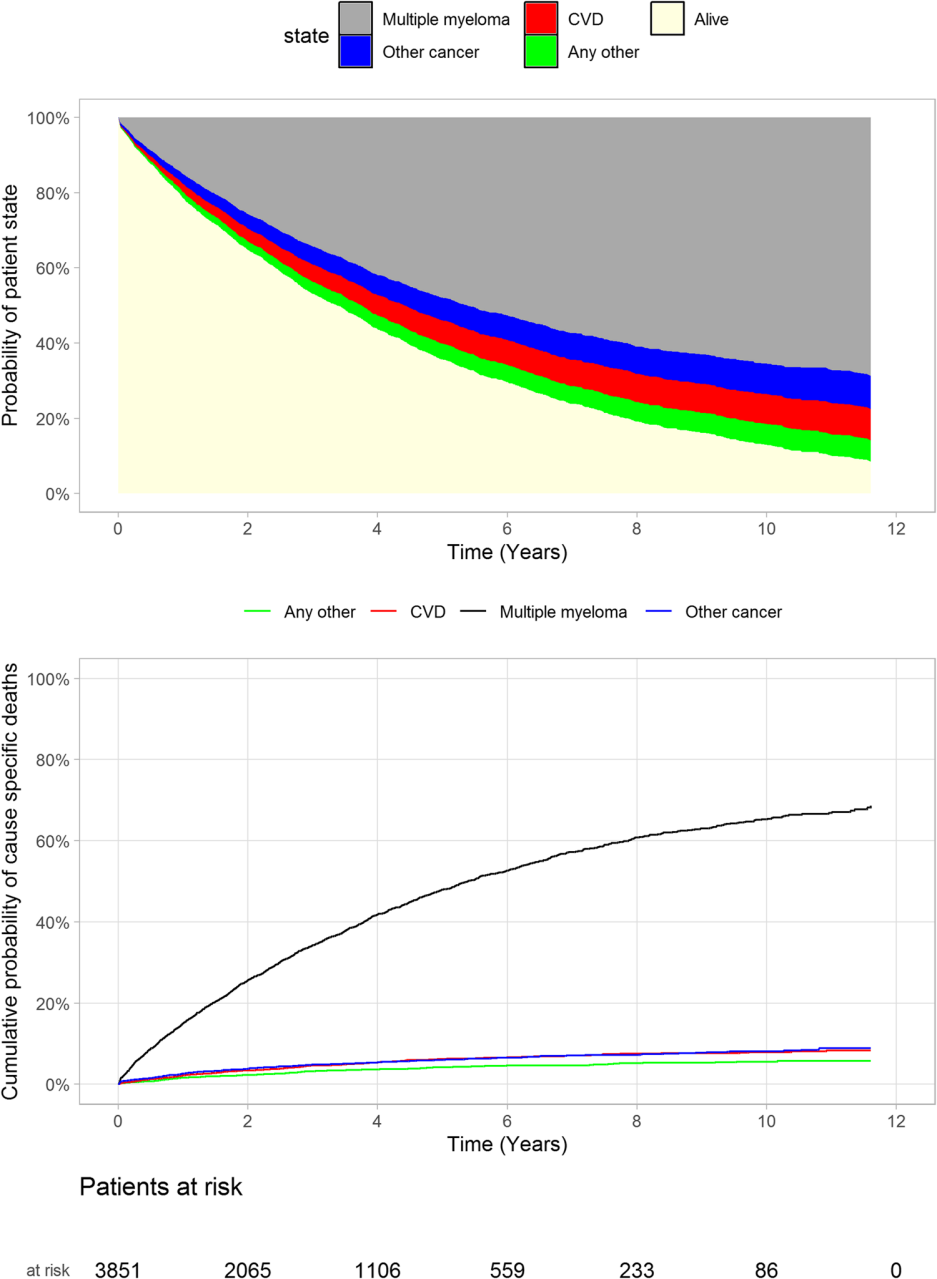
Primary causes of death and cause specific cumulative probabilities. Data is presented both as a probability of patient state (above) and cumulative probability of cause specific death (below). CVD = cardiovascular disease

Primary causes of death were accessed from Statistics Finland. The proportion of all deaths attributed to MM increased from 70.2 to 74.4% between years 1 and 4 after diagnosis (grey area), whilst the proportion of other cancer deaths reduced from 12.3 to 9.5% in the same period (Fig. 6 and Supplementary Table 5). Other cancers and CVD caused 9–10% of all annual deaths in the following years. Other cause mortality stayed relatively constant through follow-up, between 7.6 and 6.1% of all deaths. These data show that despite a significant comorbidity burden, MM remains the primary cause of death for the majority of patients with MM.

## Discussion

Accurate evaluation of the overall health status of myeloma patients is recognized as an increasingly valuable and important part of evaluating optimal treatment plans and thus ensuring the best possible treatment outcome [9]. We report a high comorbidity burden in the Finnish MM cohort at diagnosis and throughout the follow-up period, in line with previous studies. Particularly CVDs and secondary malignancies contribute to overall comorbidity load and roughly 20% of the main causes of death.

Recorded comorbidities increased rapidly within the preceding year from diagnosis, most likely due to the increasing number of investigations and healthcare visits due to MM-related symptoms [17]. Multiple myeloma can present with numerous symptoms and is often preceded by pre-malignant disease states, such as MGUS and smoldering myeloma. Positive active MM diagnosis itself requires confirmation of clonal bone marrow plasma cells exceeding 10% or biopsy-proven bony or extramedullary plasmacytoma and any one or more of the following myeloma-defining elements: hypercalcemia, renal insufficiency, anemia or bone lesions (updated criteria available at [31]). Thus, with increased MM-related healthcare contacts also many underlying comorbidities are diagnosed and recorded around MM diagnosis. From a practical perspective, a sufficient level of geriatric assessment (GA) of elderly MM patients at diagnosis is also critical to account for all relevant comorbidities that may guide treatment. A recent multicenter prospective study suggests that a comprehensive GA (CGA) is warranted in newly diagnosed elderly MM patients and may provide some added benefit to assessing patient frailty compared to the International Myeloma Working Group (IMWG) recommendations [32]. In Finland, a survey study concluded that the majority of geriatricians utilized the CGA in their general practice, but only 11% of respondents incorporated all five domains in their CGA [33]. Both the delay and misdiagnosis of both MM and comorbidities can have immediate repercussions on the treatment efficacy as well as patient outcomes.

Our CCI-stratified comorbidity data show that at study entry comorbidity burden in patients with MM was similar between the Finnish and Danish MM cohorts despite potential differences in diagnosis recording and practices between the two nations that are likely to cause some variance in the data [17]. However, differences between comorbidity classes were expected. Interestingly, although background population CVD mortality in Finnish men is among the highest in Europe, the Danish MM population had more records of CVD comorbidities compared to the Finnish MM population, whereas diabetes is more aligned with the general population in both nations [27, 28]. Although our study did not include a cohort-matched control population, a comparison of MM patients and the general population in the Danish MM nationwide study revealed the largest recorded comorbidity differences in the year preceding MM diagnosis as opposed to the past 10 years [17]. Whether the same holds true for the Finnish MM population is an intriguing addition to future studies.

Providing robust data on comorbidities in real-world patient populations to treating physicians should be emphasized as clinical trials offer insight to only a subset of patients due to strict exclusion criteria. Common examples of exclusion criteria, such as prior ASCT, prior malignancy, uncontrolled CVD or MACE or comorbid systemic illness would disqualify a significant portion of the current study population from clinical trials [34]. Although understandable due to the strict clinical trial criteria, the heterogeneity of real-world patient populations, especially predominantly diseases affecting the elderly, provides valuable information when assessing optimal treatment strategies. This is increasingly important as many MM treatments are known to have cardiotoxic effects [6, 8], and as nearly half of MM patients have a history of cardiovascular disease (Fig. 2), managing cardiovascular safety requires dynamic cooperation between cardiologists and hematologists. Additionally, some MM treatments, such as lenalidomide, are also known to have a modest effect on certain secondary malignancies, although the benefits are seen to outweigh the risks [35]. A total of 2,732 patients with MM in a large clinical trial were included in assessing second primary malignancies and concluded that the highest risk presented in transplant non-eligible patients aged > 74 years on lenalidomide maintenance therapy [15]. In phase 3, CALBG clinical trial by Holstein and colleagues, lenalidomide and placebo as maintenance treatment were compared in patients that had received a single ASCT. The trial found that cumulative secondary malignancy risk was higher in the lenalidomide arm with more haematologic, solid and non-invasive malignancies reported, but had lower risk of death from any cause [36]. In yet another study, secondary malignancy incidence and increased mortality due to these malignancies were found to be low and survival benefits afforded by lenalidomide were shown to outweigh the potential risks. Non-melanoma skin cancers accounted for 35% of all detected secondary malignancies in this study, highlighting well-known relationship between immunosuppression and skin cancer [37]. The investigators emphasise that these results warrant regular skin lesion monitoring for patients with MM on maintenance therapy. However, as with prevailing literature, the risk-to-benefit ratio remains acceptable due to significant increases in OS [35]. However, with the increased use of lenalidomide in earlier lines of treatment, the effect on secondary malignancies may become more prominent.

In comparison to clinical studies, our retrospective real-world study setting prevents us from evaluating these aforementioned risks regarding medication use and associated adverse effects. In this study, we received drug prescription information but failed to make meaningful conclusions on potential effects on comorbidity incidence due to indication, selection, and survival biases when analyzing the data, especially regarding the use of lenalidomide (data not shown). Especially since the occurrence of secondary malignancies is partially related to patient survival and intrinsic MM susceptibility, the analysis of treatment effects is better left for clinical trial-based evaluation. Other limitations of this study relate to the possible gaps in registry data and since only diagnoses from specialty health care contacts were included in this study, some comorbidity data from primary healthcare contacts could have been overlooked. The inclusion of primary health care data, a cohort-matched general population control, and a longer timeframe will provide an improvement in future studies. Despite the inherent biases of registry data, this study provided a detailed description of the comorbidity burden in patients with MM in Finland.

## Conclusions

Similar to data reported in recent epidemiological studies conducted on the MM population, Finnish patients with MM are elderly and suffer from multiple comorbidities. Although treatment practices have improved and are reflected in improved overall survival, a holistic approach accounting for diverse comorbidities is required for optimizing treatment.

## Supplementary Information

Below is the link to the electronic supplementary material.

Supplementary file1 (DOCX 45 KB)
