# Supplementary material for: Comorbidity characteristics of multiple myeloma patients diagnosed in Finland 2005–2016
Source: Ann Hematol. 2022 Sep 13;101(11):2485–95. doi: 10.1007/s00277-022-04959-9 (PMC9546994; doi:10.1007/s00277-022-04959-9)
Supplement: Supplementary file 1 — Supplementary file1 (DOCX 45 KB) [file 277_2022_4959_MOESM1_ESM.docx]

***Supplementary Information***

**Supplementary Table 1**: All recorded ICD-10 diagnoses at study entry.

| DGN | description | Overall |
| --- | --- | --- |
| n |  | 3851 |
| C90 | Multiple myeloma and malignant plasma cell neoplasms | 3851 (100.0) |
| I10 | Essential (primary) hypertension | 706 (18.3) |
| D64 | Other anaemias | 680 (17.7) |
| M54 | Dorsalgia | 450 (11.7) |
| D47 | Other neoplasms of uncertain or unknown behaviour of lymphoid, haematopoietic and related tissue | 411 (10.7) |
| I48 | Atrial fibrillation and flutter | 327 (8.5) |
| E11 | Non-insulin-dependent diabetes mellitus | 308 (8.0) |
| R70 | Elevated erythrocyte sedimentation rate and abnormality of plasma viscosity | 308 (8.0) |
| I25 | Chronic ischaemic heart disease | 256 (6.6) |
| Z01 | Other special examinations and investigations of persons without complaint or reported diagnosis | 247 (6.4) |
| N17 | Acute renal failure | 240 (6.2) |
| J18 | Pneumonia, organism unspecified | 229 (5.9) |
| Z03 | Medical observation and evaluation for suspected diseases and conditions | 210 (5.5) |
| N18 | Chronic renal failure | 209 (5.4) |
| I50 | Heart failure | 205 (5.3) |
| R10 | Abdominal and pelvic pain | 169 (4.4) |
| D48 | Neoplasm of uncertain or unknown behaviour of other and unspecified sites | 165 (4.3) |
| E78 | Disorders of lipoprotein metabolism and other lipidaemias | 147 (3.8) |
| E83 | Disorders of mineral metabolism | 135 (3.5) |
| M80 | Osteoporosis with pathological fracture | 134 (3.5) |
| R07 | Pain in throat and chest | 134 (3.5) |
| S22 | Fracture of rib(s), sternum and thoracic spine | 115 (3.0) |
| N19 | Unspecified renal failure | 113 (2.9) |
| N40 | Hyperplasia of prostate | 109 (2.8) |
| H25 | Senile cataract | 108 (2.8) |
| J45 | Asthma | 106 (2.8) |
| S32 | Fracture of lumbar spine and pelvis | 104 (2.7) |
| M48 | Other spondylopathies | 102 (2.6) |
| R06 | Abnormalities of breathing | 97 (2.5) |
| N39 | Other disorders of urinary system | 92 (2.4) |
| C61 | Malignant neoplasm of prostate | 88 (2.3) |
| D50 | Iron deficiency anaemia | 87 (2.3) |
| M17 | Gonarthrosis [arthrosis of knee] | 87 (2.3) |
| M49 | Spondylopathies in diseases classified elsewhere | 86 (2.2) |
| A41 | Other septicaemia | 80 (2.1) |
| E03 | Other hypothyroidism | 80 (2.1) |
| I20 | Angina pectoris | 77 (2.0) |
| R53 | Malaise and fatigue | 77 (2.0) |
| Z00 | General examination and investigation of persons without complaint and reported diagnosis | 77 (2.0) |
| M16 | Coxarthrosis [arthrosis of hip] | 75 (1.9) |
| R50 | Fever of other and unknown origin | 75 (1.9) |
| R91 | Abnormal findings on diagnostic imaging of lung | 75 (1.9) |
| J44 | Other chronic obstructive pulmonary disease | 74 (1.9) |
| K57 | Diverticular disease of intestine | 74 (1.9) |
| H40 | Glaucoma | 73 (1.9) |
| H90 | Conductive and sensorineural hearing loss | 73 (1.9) |
| K29 | Gastritis and duodenitis | 73 (1.9) |
| Z51 | Other medical care | 71 (1.8) |
| A49 | Bacterial infection of unspecified site | 70 (1.8) |
| S72 | Fracture of femur | 70 (1.8) |
| I35 | Nonrheumatic aortic valve disorders | 68 (1.8) |
| M51 | Other intervertebral disc disorders | 68 (1.8) |
| M79 | Other soft tissue disorders, not elsewhere classified | 68 (1.8) |
| M81 | Osteoporosis without pathological fracture | 67 (1.7) |
| N10 | Acute tubulo-interstitial nephritis | 63 (1.6) |
| Z04 | Examination and observation for other reasons | 62 (1.6) |
| J15 | Bacterial pneumonia, not elsewhere classified | 61 (1.6) |
| M84 | Disorders of continuity of bone | 60 (1.6) |
| M90 | Osteopathies in diseases classified elsewhere | 60 (1.6) |
| Z71 | Persons encountering health services for other counselling and medical advice, not elsewhere classified | 59 (1.5) |
| I21 | Acute myocardial infarction | 57 (1.5) |
| H35 | Other retinal disorders | 56 (1.5) |
| S42 | Fracture of shoulder and upper arm | 56 (1.5) |
| G47 | Sleep disorders | 55 (1.4) |
| C50 | Malignant neoplasm of breast | 54 (1.4) |
| E87 | Other disorders of fluid, electrolyte and acid-base balance | 53 (1.4) |
| C44 | Other malignant neoplasms of skin | 52 (1.4) |
| I63 | Cerebral infarction | 51 (1.3) |
| R52 | Pain, not elsewhere classified | 51 (1.3) |
| Z95 | Presence of cardiac and vascular implants and grafts | 51 (1.3) |
| C91 | Lymphoid leukaemia | 50 (1.3) |
| I26 | Pulmonary embolism | 48 (1.2) |
| N08 | Glomerular disorders in diseases classified elsewhere | 47 (1.2) |
| K59 | Other functional intestinal disorders | 45 (1.2) |
| N30 | Cystitis | 44 (1.1) |
| R31 | Unspecified haematuria | 44 (1.1) |
| A40 | Streptococcal septicaemia | 43 (1.1) |
| M35 | Other systemic involvement of connective tissue | 43 (1.1) |
| C92 | Myeloid leukaemia | 42 (1.1) |
| D12 | Benign neoplasm of colon, rectum, anus and anal canal | 42 (1.1) |
| M75 | Shoulder lesions | 42 (1.1) |
| K80 | Cholelithiasis | 41 (1.1) |
| C41 | Malignant neoplasm of bone and articular cartilage of other and unspecified sites | 40 (1.0) |
| D46 | Myelodysplastic syndromes | 40 (1.0) |
| D69 | Purpura and other haemorrhagic conditions | 40 (1.0) |
| K25 | Gastric ulcer | 40 (1.0) |
| R33 | Retention of urine | 40 (1.0) |
| T81 | Complications of procedures, not elsewhere classified | 40 (1.0) |
| R04 | Haemorrhage from respiratory passages | 39 (1.0) |
| C79 | Secondary malignant neoplasm of other sites | 38 (1.0) |
| Z50 | Care involving use of rehabilitation procedures | 38 (1.0) |
| I34 | Nonrheumatic mitral valve disorders | 37 (1.0) |
| I80 | Phlebitis and thrombophlebitis | 37 (1.0) |

**Supplementary Table 2:** Charlson comorbidity index (CCI) comorbidities recorded in cohort prior to MM diagnosis – MM patient comorbidities by CCI class compared to 2005-2012 Danish National Multiple Myeloma Registry

| Diagnosis | ICD-10 codes | This study –  3851 Patients with MM  Number (%) | Gregersen study [17] - 2190 Patients with MM  Number (%) | Significance (p) |
| --- | --- | --- | --- | --- |
| Any CCI COMORBIDITY | Any ICD-10 code below | 1464 (38.0) | 896 (40.9) | 0.028 |
| Myocardial infarction | I21.x, I22.x, I25.2 | **93 (2.4)** | 118 (5.4) | **<0.001** |
| Congestive heart failure | I09.9, I11.0, I13.0, I13.2, I25.5, I42.0, I42.5 - I42.9, I43.x, I50.x, P29.0 | 220 (5.7) | 126 (5.8) | 0.954 |
| Peripheral vascular disease | I70.x, I71.x, I73.1, I73.8, I73.9, I77.1, I79.0, I79.2, K55.1, K55.8, K55.9, Z95.8, Z95.9 | **59 (1.6)** | 82 (3.7) | **<0.001** |
| Cerebrovascular disease | G45.x, G46.x, H34.0, I60.x - I69.x | **116 (3)** | 160 (7.3) | **<0.001** |
| Dementia | F00.x - F03.x, F05.1, G30.x, G31.1 | 60 (1.6) | **17 (0.8)** | **0.009** |
| Chronic pulmonary disease | I27.8, I27.9, J40.x - J47.x, J60.x - J67.x, J68.4, J70.1, J70.3 | **183 (4.8)** | 147 (6.7) | **0.001** |
| Rheumatic disease | M05.x, M06.x, M31.5, M32.x - M34.x, M35.1, M35.3, M36.0 | 98 (2.5) | 72 (3.3) | 0.105 |
| Peptic ulcer disease | K25.x - K28.x | **49 (1.3)** | 89 (4.1) | **<0.001** |
| Mild liver disease | B18.x, K70.0 - K70.3, K70.9, K71.3 - K71.5, K71.7, K73.x, K74.x, K76.0, K76.2 - K76.4, K76.8, K76.9, Z94.4 | 19 (0.5) | 17 (0.8) | 0.223 |
| Diabetes without chronic complication | E10.0, E10.1, E10.6, E10.8, E10.9, E11.0, E11.1, E11.6, E11.8, E11.9, E12.0, E12.1, E12.6, E12.8, E12.9, E13.0, E13.1, E13.6, E13.8, E13.9, E14.0, E14.1, E14.6, E14.8, E14.9 | 239 (6.2) | **71 (3.2)** | **<0.001** |
| Diabetes with chronic complication | E10.2 - E10.5, E10.7, E11.2 - E11.5, E11.7, E12.2 - E12.5, E12.7, E13.2 - E13.5, E13.7, E14.2 - E14.5, E14.7 | **80 (2)** | 73 (3.3) | **0.004** |
| Hemiplegia or paraplegia | G04.1, G11.4, G80.1, G80.2, G81.x, G82.x, G83.0 - G83.4, G83.9 | 30 (0.8) | **7 (0.3)** | **0.026** |
| Renal disease | I12.0, I13.1, N03.2 - N03.7, N05.2 - N05.7, N18.x, N19.x, N25.0, Z49.0 - Z49.2, Z94.0, Z99.2 | 303 (7.9) | **131 (6.0)** | **0.006** |
| Any malignancy, including lymphoma and leukaemia, except malignant neoplasm of skin (C44) and Multiple myeloma (C90) | C00.x - C26.x, C30.x - C34.x, C37.x - C41.x, C43.x, C45.x - C58.x, C60.x - C76.x, C81.x - C85.x, C88.x, C90.x - C97.x | 481 (12.5) | 252 (11.5) | 0.803 |
| Moderate or severe liver disease | I85.0, I85.9, I86.4, I98.2, K70.4, K71.1, K72.1, K72.9, K76.5, K76.6, K76.7 | 6 (0.2) | 2 (0.1) | 0.719 |
| Metastatic solid tumour | C77.x - C80.x | 54 (1.4) | 41 (1.9) | 0.163 |
| AIDS/HIV | B20.x - B22.x, B24.x | <5 (masked) | 0 (0.0) | 1.000 |

**Supplementary Table 3**: All recorded malignancies (N >= 5) at study entry.

| DGN | description | N | % of Patients with MM | % of all malignancies |
| --- | --- | --- | --- | --- |
| n |  | 3851 | 3851 | 742 |
| C61 | Malignant neoplasm of prostate | 88 | 2.3 | 12.7 |
| C50 | Malignant neoplasm of breast | 54 | 1.4 | 7.8 |
| C44 | Other malignant neoplasms of skin | 52 | 1.4 | 7.5 |
| C91 | Lymphoid leukaemia | 50 | 1.3 | 7.2 |
| C92 | Myeloid leukaemia | 42 | 1.1 | 6.0 |
| C41 | Malignant neoplasm of bone and articular cartilage of other and unspecified sites | 40 | 1 | 5.8 |
| D46 | Myelodysplastic syndromes | 40 | 1 | 5.8 |
| D47 | Other neoplasms of uncertain or unknown behaviour of lymphoid, haematopoietic and related tissue | 38 | 1 | 5.5 |
| C96 | Other and unspecified malignant neoplasms of lymphoid, haematopoietic and related tissue | 23 | 0.6 | 3.3 |
| C64 | Malignant neoplasm of kidney, except renal pelvis | 22 | 0.6 | 3.2 |
| C67 | Malignant neoplasm of bladder | 21 | 0.5 | 3.0 |
| C83 | Diffuse non-Hodgkin's lymphoma | 20 | 0.5 | 2.9 |
| D41 | Neoplasm of uncertain or unknown behaviour of urinary organs | 19 | 0.5 | 2.7 |
| C18 | Malignant neoplasm of colon | 16 | 0.4 | 2.3 |
| C20 | Malignant neoplasm of rectum | 16 | 0.4 | 2.3 |
| C88 | Malignant immunoproliferative diseases | 14 | 0.4 | 2.0 |
| D32 | Benign neoplasm of meninges | 14 | 0.4 | 2.0 |
| C95 | Leukaemia of unspecified cell type | 13 | 0.3 | 1.9 |
| C85 | Other and unspecified types of non-Hodgkin's lymphoma | 12 | 0.3 | 1.7 |
| D45 | Polycythaemia vera | 12 | 0.3 | 1.7 |
| C43 | Malignant melanoma of skin | 11 | 0.3 | 1.6 |
| C49 | Malignant neoplasm of other connective and soft tissue | 11 | 0.3 | 1.6 |
| D43 | Neoplasm of uncertain or unknown behaviour of brain and central nervous system | 11 | 0.3 | 1.6 |
| C34 | Malignant neoplasm of bronchus and lung | 10 | 0.3 | 1.4 |
| C54 | Malignant neoplasm of corpus uteri | 9 | 0.2 | 1.3 |
| C73 | Malignant neoplasm of thyroid gland | 9 | 0.2 | 1.3 |
| C76 | Malignant neoplasm of other and ill-defined sites | 9 | 0.2 | 1.3 |
| C40 | Malignant neoplasm of bone and articular cartilage of limbs | 8 | 0.2 | 1.2 |
| C93 | Monocytic leukaemia | 6 | 0.2 | 0.9 |
| C81 | Hodgkin's disease | 5 | 0.1 | 0.7 |

**Supplementary Table 4:** Most common incident secondary malignancies recorded during follow-up

| Diagnosis | ICD-10 description | Overall - n (%) |
| --- | --- | --- |
| C91 | Lymphoid leukaemia | 115 (3.6) |
| C44 | Other malignant neoplasms of skin | 77 (2.4) |
| C92 | Myeloid leukaemia | 65 (2.0) |
| C61 | Malignant neoplasm of prostate | 47 (1.5) |
| D46 | Myelodysplastic syndromes | 33 (1.0) |
| C50 | Malignant neoplasm of breast | 32 (1.0) |
| C83 | Diffuse non-Hodgkin's lymphoma | 26 (0.8) |
| C34 | Malignant neoplasm of bronchus and lung | 22 (0.7) |
| C96 | Other and unspecified malignant neoplasms of lymphoid, haematopoietic and related tissue | 21 (0.7) |
| D41 | Neoplasm of uncertain or unknown behaviour of urinary organs | 21 (0.7) |
| C09 | Malignant neoplasm of tonsil | 17 (0.5) |
| C18 | Malignant neoplasm of colon | 17 (0.5) |

**Supplementary Table 5:** Timepoint estimates for the primary causes of death through follow-up

| Time (years) | Primary cause of death | |  |  |
| --- | --- | --- | --- | --- |
|  | MM | Other cancer | CVD | Other causes |
| 0 | 0 | 0 | 0 | 0 |
| 1 | 70.2% | 12.3% | 9.9% | 7.6% |
| 2 | 73.0% | 10.9% | 9.7% | 6.5% |
| 3 | 73.1% | 10.3% | 9.8% | 6.9% |
| 4 | 74.4% | 9.5% | 9.6% | 6.5% |
| 5 | 74.5% | 9.3% | 9.6% | 6.5% |
| 6 | 74.7% | 9.3% | 9.4% | 6.5% |
| 7 | 75.3% | 9.3% | 9.3% | 6.1% |
| 8 | 75.3% | 9.0% | 9.3% | 6.4% |
| 9 | 75.2% | 9.3% | 9.1% | 6.4% |
| 10 | 75.2% | 9.4% | 9.1% | 6.4% |
| 11 | 74.5% | 9.9% | 9.2% | 6.4% |
| 12 | 75.0% | 9.7% | 9.0% | 6.3% |
